# Research progress on microRNA-1258 in the development of human cancer

**DOI:** 10.3389/fonc.2022.1024234

**Published:** 2022-09-29

**Authors:** Mengjia Qian, Yuke Xia, Gong Zhang, Han Yu, Yiyao Cui

**Affiliations:** Department of Thyroid and Breast Surgery, The Affiliated JiangNing Hospital of Nanjing Medical University, Nanjing, China

**Keywords:** miR-1258, cancer, tumor suppressor, biological function, clinical application

## Abstract

microRNAs (miRNAs) are small endogenous RNAs composed of 20-22 nucleotides that do not encode proteins, which regulate the expression of downstream genes by targeting the 3’ untranslated region of mRNA. Plentiful research has demonstrated that miRNAs participate in the initiation and development of diverse diseases and malignant tumors. miR-1258 exerts great influence on tumors, including tumor growth, distant metastasis, migration, invasion, chemosensitivity, cell glycolysis, apoptosis, and stemness. Interestingly, miR-1258 is a miRNA with explicit functions and has been investigated to act as a tumor suppressor in studies on various types of tumors. With accumulating research on miR-1258, it has been found to be used as a biomarker in the early diagnosis and prognosis prediction of tumor patients. In this review, we outline the development of miR-1258 research, describe its regulatory network, and discuss its roles in cancer. Additionally, we generalize the potential clinical applications of miR-1258. This review offers emerging perspectives and orientations for further comprehending the function of miR-1258 as a diagnostic and prognostic biomarker and potent therapeutic target in cancer.

## Introduction

MicroRNAs (miRNAs) are non-coding RNAs composed of about 21-25 nucleotides, which are widely distributed from viruses to numerous cells (1). These miRNAs generally target one or more messenger RNAs (mRNAs), split them directly, or block their translation by binding to mRNAs, thereby blocking their protein production (2).

miRNAs are produced by endogenous transcription of primary transcripts, first cut in the nucleus by Drosha, generating stem-loop precursor miRNAs (about 70 nucleotides). Subsequently, Exportin5 transports precursor miRNA from the nucleus to the cytoplasm. Finally, mature miRNAs about 22 nucleotides in length are processed by Dicer (3).

Heretofore, most of the miRNAs found and reported are highly conserved among species, which are closely related to the significance of their functions (4). miRNAs play an essential role in the regulation of cell differentiation, tissue development, metabolism, and tumorigenesis (5, 6). Extensive studies have shown that miRNAs are dysregulated in tumors, and they widely participate in the whole process of tumor development as tumor suppressors or carcinogens (7–11). Moreover, miRNAs also act key roles in early diagnosis, treatment response predictors, and prognostic biomarkers of cancer (12–16).

miR-1258 is located in the first intron of its host gene ZNF385B (Zinc Finger Protein 385B) on chromosome 2q31.3 and plays a key regulatory role in intestinal barrier function, herpesvirus Lytic replication, bronchopulmonary dysplasia, brown adipose differentiation, and other diseases (17–20). miR-1258 is a miRNA with explicit functions and has been reported to act as a tumor suppressor in studies on diverse types of tumors. So far, there is no review on the research progress of miR-1258 in the development of human cancer. Hence, we first comprehensively and systematically reviewed the research progress of miR-1258 in the inhibitory role of human cancer and its detailed mechanism, to better translate the key role of miR-1258 into diagnostic and prognostic biomarkers and potential targets of cancer.

## miR-1258 expression in human cancer

As shown in **Table 1**, miR-1258 was widely downregulated in the tumor tissues and cell lines of hepatocellular carcinoma (HCC) (21–25), gastric cancer (GC) (26), colorectal cancer (CRC) (27–29), oral squamous cell carcinoma (OSCC) (30), esophageal cancer (EC) (31), non-small cell lung cancer (NSCLC) (32–34), breast cancer (BC) (35–40), cervical cancer (CC) (41), myeloma (42), thyroid carcinoma (43), glioblastoma (44), and osteosarcoma (45) *via* the detection of qRT-PCR. In addition, the hypermethylation of the CpG island of the miR-1258 host gene was detected in ovarian cancer (OC), myeloma, prostate cancer, and BC *via* the methylation-specific PCR analysis (38, 42, 46–49). Since 2011, miR-1258 has been widely investigated and reported as a tumor suppressor.

**Table 1:** Expression profiles of miR-1258 in human cancers.

Systems	RNAs	Cancer type	Role	Expression	Sources	Sample number	Biological functions	Targets	Upstream gene	References
Digestive system	miR-1258	HCC	tumor suppressor	downregulation	tissues and cell lines	38 paired	Inhibit cell proliferation, migration, invasion and glycolysis process	RPN2	Circ_0046599	[21]
	miR-1258			downregulation	tissues and cell lines	37 paired	Inhibit cell proliferation, cell cycle transition, migration, invasion, EMT and facilitate cell apoptosis	SERBP1	Circ_0046600	[22]
	miR-1258			/	/	/	Inhibit cell migration and invasion	TCF4	Kindlin-2	[23]
	miR-1258			downregulation	tissues and cell lines	20 paired	Inhibit cell proliferation, cell cycle transition, migration, stemness, facilitate cell apoptosis and increase chemosensitivity	CKS1B	/	[24]
	miR-1258			downregulation	tissues and cell lines	20 paired	Inhibit cell migration and invasion	Smad2/3	LINC01278	[25]
	miR-1258	GC	tumor suppressor	downregulation	tissues and cell lines	116 paired	Inhibit cell invasion and metastasis	HPSE		[26]
	miR-1258	CRC	tumor suppressor	downregulation	cell lines	/	Inhibit cell proliferation and migration	RPN2	circ_SMAD2	[27]
	miR-1258			downregulation	tissues and cell lines	60 paired	Inhibit cell proliferation and arrest cell cycle transition	E2F8	/	[28]
	miR-1258			/	/	/	Inhibit cell proliferation and migration	CKS1B	/	[29]
	miR-1258	OSCC	tumor suppressor	downregulation	tissues	89 paired	Inhibit cell proliferation and invasion	SP1	c‐Myb	[30]
	miR-1258	EC	tumor suppressor	downregulation	tissues	40 paired	Inhibit cell proliferation, migration and invasion	/	LncRNA ASB16-AS1	[31]
Respiratory system	miR-1258	NSCLC	tumor suppressor	downregulation	tissues and cell lines	50 paired	Inhibit cell proliferation, cell cycle transition and induce cell senescence and apoptosis	GRB2	/	[32]
	miR-1258		tumor suppressor	downregulation	tissues	53 paired	Inhibit cell invasion	/	/	[33]
	miR-1258		tumor suppressor	downregulation	serum and cell lines	96	Inhibit cell proliferation, migration and invasion	RHOV	circ_0000519	[34]
Genitourinary system	miR-1258	BC	tumor suppressor	downregulation	tissues	83 paired	Inhibit cell proliferation, migration and stemness	KDM7A	circ_002178	[35]
	miR-1258		biomarker	downregulation	tissues	1166	prognostic biomarker	/	/	[36]
	miR-1258		tumor suppressor	downregulation	cell lines	/	Inhibit cell proliferation, cell cycle transition and induce cell apoptosis	E2F1	/	[37]
	miR-1258		biomarker	downregulation	tissues	239	prognostic biomarker	/	/	[38]
	miR-1258		tumor suppressor	downregulation	tissues and cell lines	13 paired	Inhibit cell invasion and experimental brain metastasis	HPSE		[40]
	miR-1258	CC	tumor suppressor	downregulation	cell lines	/	Inhibit cell proliferation, invasion, migration and promote cell apoptosis	E2F1	/	[41]
Other	miR-1258	Myeloma	tumor suppressor	downregulation	tissues	113	Inhibit cell proliferation and promote cell apoptosis	PD-L1	/	[42]
	miR-1258	Thyroid carcinoma	tumor suppressor	downregulation	cell lines	/	Inhibit the cell viability, migration and invasion	TMPRSS4	/	[43]
	miR-1258	Glioblastoma	tumor suppressor	downregulation	tissues	38	Inhibit cell proliferation, migration, invasion and increase chemosensitivity	E2F1	/	[44]
	miR-1258	Osteosarcoma	tumor suppressor	downregulation	tissues and cell lines	60 paired	Inhibit cell proliferation and cell cycle transition	AKT3	/	[45]

HCC, hepatocellular carcinoma; OSCC, oral squamous cell carcinoma; CRC, colorectal cancer; EC, esophageal cancer; NSCLC, non-small cell lung cancer; BC, breast cancer; CC, cervical cancer; GC, gastric cancer.

## The regulatory network of miR-1258 in cancer

**Figure 1:**
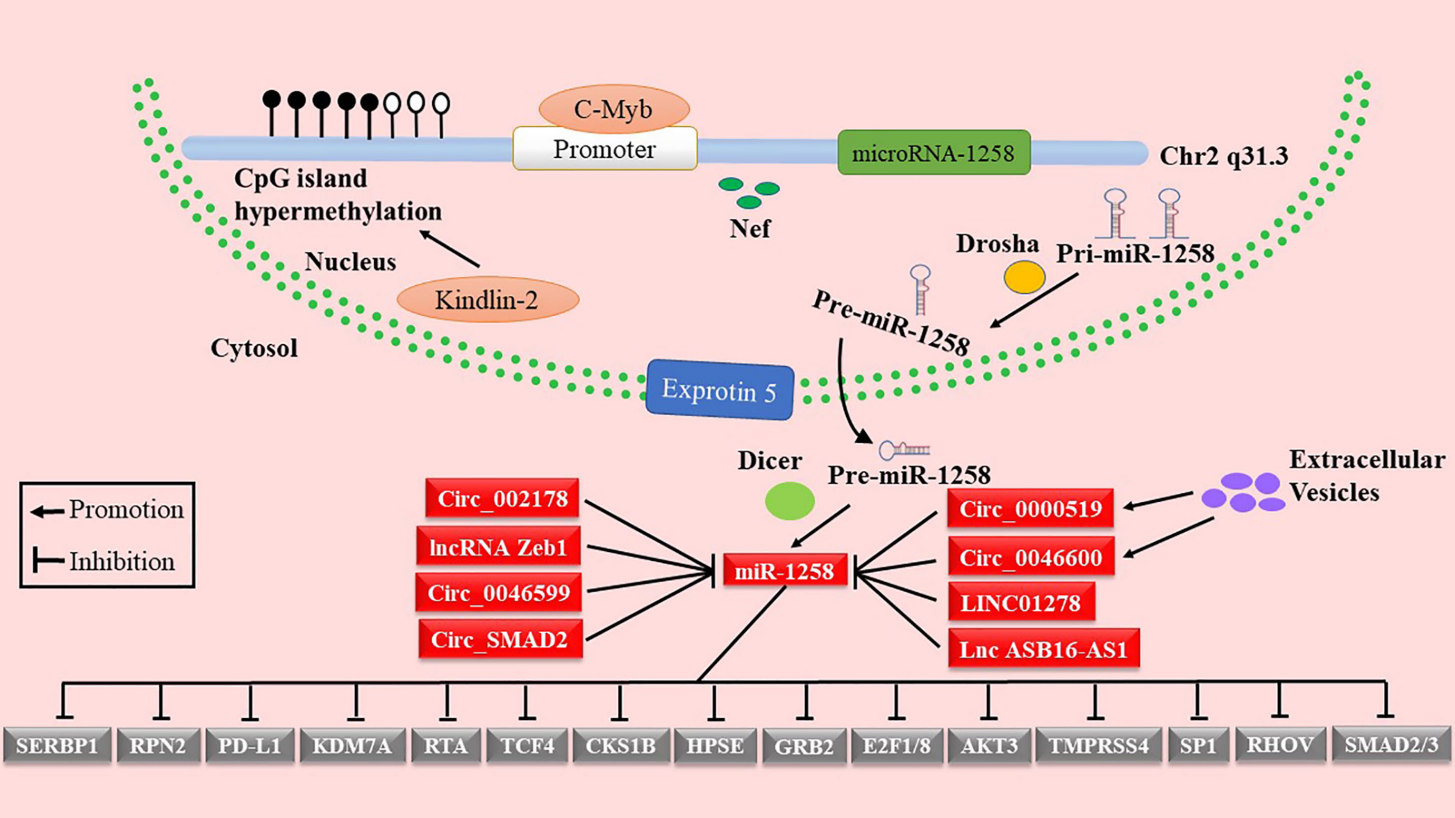
Mechanism and function of miR-1258 downregulation in human cancer.

As shown in **Figure 1** and **Table 1**, miR-1258 was regulated by Nef, c-Myb, and Kindlin-2 at the transcriptional stage. Yan et al. reported that negative factor (Nef), a secreted HIV-1 protein, elevated the expression of has-miR-1258 in primary effusion lymphoma cells (18). Kindlin-2 inhibited the transcription of miR-1258 by increasing methylation of the CpG island in the miR-1258 promoter (23). Moreover, c-Myb, a transcriptional factor, is directly bound to the promoter of has-miR-1258 to repress the transcription of miR-1258 (30). Furthermore, the hypermethylation of the CpG island of the miR-1258 promoter was demonstrated in the tissues of ovarian cancer, myeloma, prostate cancer, and BC, inhibiting the transcription of miR-1258 (38, 42, 46–49).

Accumulative studies indicated that the mature miRNAs were inactivated by lncRNAs or circRNAs through competing endogenous RNAs (ceRNAs) (50–52). miR-1258 was inactivated by LncRNA Zeb1 in the intestinal barrier (19). In addition, miR-1258 was targeted by circ_0046599, circ_0046600, and lncRNA LINC01278 in HCC (21, 22, 25). Besides, extracellular vesicles also acted key roles in the regulation of miR-1258 by transferring circ_0000519 as ceRNA in NSCLC (34). Zhang et al. noted that upregulated circ_SMAD2 suppressed the expression of miR-1258 through the ceRNAs mechanism in CRC (27). LncRNA ASB16-AS1, as sponge molecules, regulated the EC progression by absorbing miR-1258 (31). Li et al. reported that miR-1258 was directly regulated by circ_002178 *via* using luciferase reporter assay (35). Therefore, it is necessary to provide a theoretical basis for using miR-1258 as a potential therapeutic target by in-depth excavating of the regulatory mechanisms of miR-1258 in cancer.

## The biological functions of miR-1258 in cancer

**Figure 2:**
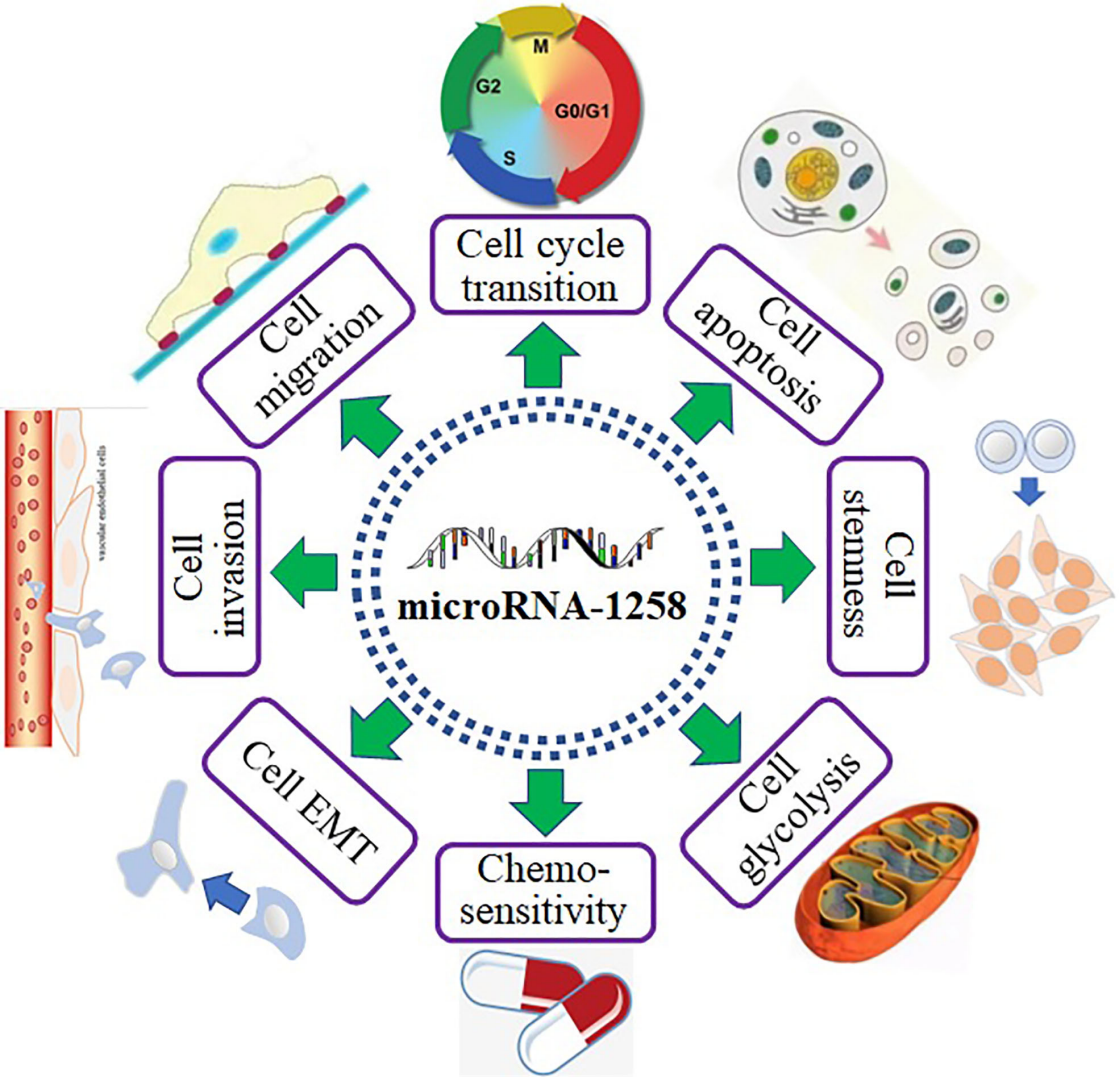
The biological functions of miR-1258 in human cancer.

MiR-1258 profoundly inhibited tumor progression by binding to the mRNA of downstream genes (**Figure 1**). As shown in **Figure 2**, miR-1258 was involved in the biological processes of the cell cycle transition, cell apoptosis, cell stemness, cell migration and invasion, and EMT to restrain the progression of tumors. Also, miR-1258 repressed glycolysis metabolisms by targeting the mRNA of RPN2 to suppress the cell growth of HCC (21). The stemness of cancer cells plays a crucial role in the survival, proliferation, recurrence, and drug resistance of cancer (53). miR-1258 significantly suppressed the cell stemness and tumor progression of HCC by binding to the mRNA of cyclin-dependent kinase regulatory subunit 1B (CKS1B) (24). In the following parts, we comprehensively revealed the biological functions of miR-1258 as tumor suppressors.

## Inhibition of the cancerogenic process in human cancer

**Figure 3:**
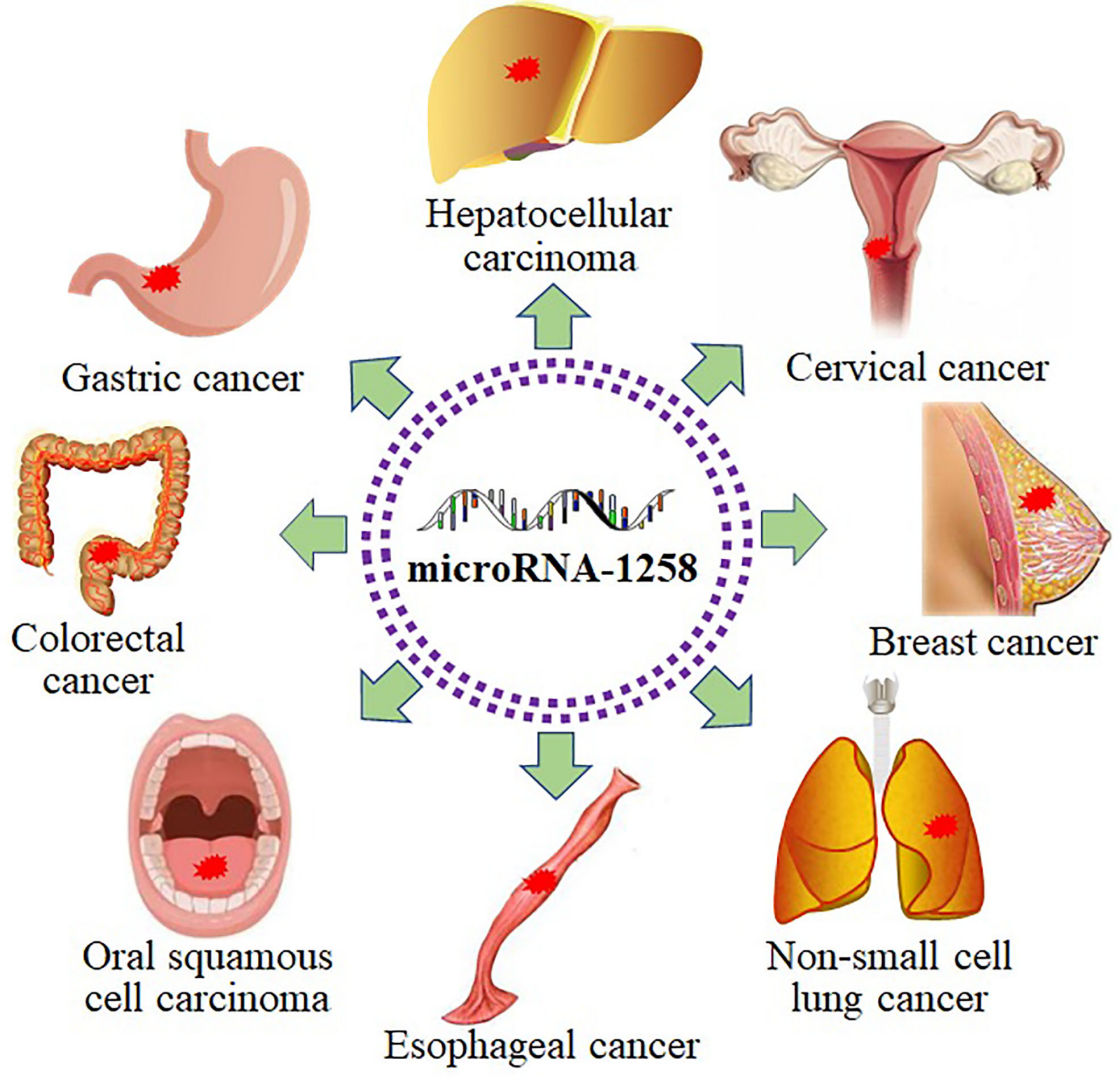
MiR-1258 functions as a tumor suppressor in diverse cancer.

MiR-1258 was extensively investigated in digestive system-related tumors as a tumor suppressor, including HCC (21–25), GC (26), CRC (27–29), OSCC (30), and EC (31). MiR-1258 inhibited cell viability and tumor progression by targeting key signaling pathway proteins and related transcription factors to function as a tumor suppressor in gastrointestinal cancer. Tumor cells are characterized by immortal and infinitely dividing cells (54). Overexpressed miR-1258 induced cell senescence and apoptosis and suppressed cell viability to mitigate tumor progression by binding to the targets in NSCLC (32–34). As a histone demethylase, KDM7A extensively affects the malignant biological behaviors of tumor cells by regulating cell cycle transition (55). The progression of BC was prohibited by miR-1258 through increasing cell apoptosis and stemness and decreasing cell viability and cell cycle transition *via* devitalizing key proteins, including KDM7A (35–38, 40). Furthermore, miR-1258 also functioned as a tumor suppressor in CC by restraining cell proliferation and enhancing cell apoptosis *via* targeting E2F1 (41). Besides, miR-1258 still repressing the tumor process in myeloma, thyroid carcinoma, glioblastoma, and osteosarcoma (42–45). Abovementioned facts indicated that miR-1258 was extensively involved in the development of human cancer (**Figure 3**).

## Promotion of cell apoptosis and chemosensitivity

Currently, numerous small-molecule anti-cancer drugs are targeting molecules involved in cell apoptosis (56). Growth factor receptor binding protein 2 (GRB2) is a key adapter protein that activates the RAS/ERK signaling pathway, and its dysregulation can profoundly affect the process of cell apoptosis in cancer (57). miR-1258 inhibited the NSCLC progression *via* inducing cell apoptosis and senescence by directly targeting GRB2 (32). Tumor cells evade immune clearance by increasing the expression of PD-L1 on the surface and inhibiting T cell function *via* binding to PD-1 on the surface of T cells (58). Wang et al. reported that overexpressed miR-1258 inhibited cell proliferation and increased cell apoptosis by restraining the expression of PD-L1 in completely methylated myeloma cells (42). In addition, miR-1258 strengthened the cell apoptosis to repress cell proliferation by binding to mRNA of SERBP1, CKS1B, and E2F1 in HCC (22, 24) and BC (37). Furthermore, the sensitivity of chemotherapy was increased by enhancing cell apoptosis (59). Hu et al. indicated that upregulated miR-1258 greatly reinforced the sensitivity of HCC cells to chemotherapy drugs *in vivo* by restraining the expression of CKS1B (24).

## Suppression of cell migration and invasion

The migratory and invasive ability of cells largely determines whether distant metastasis occurs in tumors, which is the main reason for the poor prognosis of patients with cancer (60). TGF-β (transforming growth factor-β)/Smad signaling pathway significantly regulates the biological behaviors of cell migration and invasion in cancer (61). Huang et al. found that miR-1258 inhibited the metastasis of cells by impairing the migratory and invasive ability of cells *via* targeting the mRNA of Smad2 and Smad3 in HCC (25). Besides, the translation of RPN2, TCF4, KDM7A, HPSE, TMPRSS4, and E2F1 was also suppressed by miR-1258 overexpression, according to recent research, which prevented cell invasion and migration in HCC (21, 23), CRC (27), BC (35, 40), and TC (43). It is impossible to disregard the roles of the matrix metalloproteinase (MMP) family in regulating cell migration and invasion (62). Qin et al. demonstrated that miR-1258 inhibited the transcription of MMP2 and PCNA through binding to the mRNA of E2F1 to depress the cell migration and invasion in glioblastoma (44). The abovementioned data indicated that miR-1228 regulated cell migration and invasion by mainly interacting with the TGF-β/Smad pathway and MMP family.

## Inhibition of cell cycle transition

The cell cycle includes four consecutive phases of G0/G1, S, G2, and M, which is a set of organized and monitored events that are responsible for dividing cells into two daughter cells. The aberrant regulation of cell cycle transition has played a critical role in the growth and development of tumors (63). E2F family performs crucial functions in controlling cell cycle, maintaining genomic integrity, and coping with replication stress and DNA damage as transcriptional factors (64). miR-1258 arrested the cell cycle in G0/G1 phase by targeting E2F1 and E2F8 in CRC, BC, CC, and glioblastoma (28, 37, 41, 44). CKS1B is engaged in the transcription regulation of a series of genes involved in the cell cycle process, which is closely related to the abnormal cell proliferation of tumors (65). Overexpressed miR-1258 greatly inhibited cell cycle arrest in G0/G1 phase by directly repressing the expression of CKS1B in HCC (24). Additionally, miR-1258 inhibited the cell cycle transition by binding to mRNA of SERBP1, GRB2, and AKT3 in HCC (22), NSCLC (32), and osteosarcoma (45), respectively. These data proved that miR-1258, a potential therapeutic target, performed key roles in cell cycle arrest in cancer.

## Role in EMT

Epithelial-mesenchymal transition (EMT) refers to the transformation of epithelial cells into invasive mesenchymal cells, which plays a crucial function in the invasion and metastasis of various types of cancer (66, 67). SP1, as a transcription factor, directly modulates the EMT and metastasis of cancer at transcriptional levels (68, 69). Overexpressed miR-1258 significantly repressed the EMT and metastasis in OSCC cells by targeting the mRNA of SP1 (30). In addition, Lin et al. demonstrated that miR-1258 suppressed the EMT and metastasis of HCC cells through targeting TCF4, a key member of the Wnt/β-catenin signaling pathway (23). Heparanase (HPSE) is a potent enzyme that fosters tumor growth, angiogenesis, and metastasis (70). Its dysregulation can produce a wide range of effects that significantly alter the microenvironment, stimulating cell growth and metastasis of tumors (71). Zhang et al. first revealed that upregulated miR-1258 inhibited breast cancer brain metastasis through targeting HPSE by using regulatory experiments, functional experiments, and clinical specimens’ validation (40). Moreover, overexpression of miR-1258 suppressed the cell metastasis through repressing the expression of RHOV in NSCLC both *in vitro* and *in vivo (* 34). Collectively, miR-1258 can effectively regulate the EMT process and metastasis through targeting different genes at the post-transcriptional level in cancer.

## Clinical application

It has been reported that it is promising to manipulate these miRNAs for cancer treatment by combining effective applications of miRNA delivery systems, such as chemical modification of miRNAs, lipid-based miRNA delivery systems, and organic/inorganic composite nanoparticles (72). Besides, abundant studies have found that the differential profiles of miRNAs in circulation or tissues were closely correlated to the early diagnosis, clinical stage, response to therapy, and pathological characteristics of tumors (11). In addition, the abnormal expression of miRNAs can also be used to predict the long-term survival of tumor patients (73). The probability of distant metastasis and the clinical stage of OC can be predicted by the frequency of methylation in the promoter of the miR-1258 host gene. Metastatic patients had a twofold higher rate of miR-1258 methylation than non-metastatic OC patients did (46, 47). In addition, the methylation level of miR-1258 was positively correlated to the advanced clinical stage and pathological characteristics of OC, BC, and myeloma (38, 42, 48). Besides, the level of miR-1258 promoter methylation can accurately diagnose prostate cancer in clinical samples with 97.8% sensitivity and 100% specificity (49). Due to the important role of miR-1258 as a tumor suppressor, the level of its expression profoundly affected the prognosis and clinicopathological characteristics of tumor patients. As shown in **Table 2**, a lower level of miR-1258 meant an inferior progression-free survival (PFS) and a higher probability of recurrence in 63 patients of myeloma (42). Low expression of miR-1258 was not only associated with the advanced clinical stage but also meant worse overall survival (OS) and relapse-free survival (RFS) in BC patients (36, 39). In addition, the level of miR-1258 was negatively correlated with the probability of tumor recurrence and metastasis and poor disease-free survival (DFS), OS, and RFS in HCC (24, 25). Shi et al. demonstrated that miR-1258 was negatively correlated to advanced clinical stage and lymphatic vessel invasion by analyzing the postoperative pathological data of 116 GC patients (26). Furthermore, a lower expression of miR-1258 in ESCC patients meant a shorter OS and DFS (30). Qin et al. discovered that the level of miR-1258 expression was decreased with the elevation of pathological grade by analyzing the postoperative pathological results of 33 glioblastoma patients (44). In addition, patients with low miR-1258 were greatly related to bigger tumor size in CRC (28). The OS would be greatly shortened in osteosarcoma patients with a low level of miR-1258 expression. Meanwhile, decreased miR-1258 was strongly correlated to the malignant clinicopathological characteristics of patients with osteosarcoma (45). These results verified the significant role and prospective clinical relevance of miR-1258 as diagnostic and prognostic biomarkers in cancer.

**Table 2:** Clinical implication of miR-1258 in human cancers.

Cancertype	Sources	Sample number	Aggressive phenotype of low miR-1258	Prognosis of low miR-1258	References
HCC	Tissues	20	Yes	Worse DFS	[24]
HCC	Tissues	20	/	Worse OS and RFS	[25]
GC	Tissues	116	Yes	/	[26]
CRC	Tissues	60	Yes	/	[28]
OSCC	Tissues	89	/	Worse OS and DFS	[30]
BC	Tissues	1062	Yes	Worse OS	[36]
BC	Tissues	105	Yes	Worse OS and RFS	[39]
Myeloma	Tissues	63	/	Worse PFS	[42]
Glioblastoma	Tissues	33	Yes	/	[44]
Osteosarcoma	Tissues	60	Yes	Worse OS	[45]

BC, breast cancer; HCC, hepatocellular carcinoma; GC, gastric cancer; OSCC, oral squamous cell carcinoma; CRC, colorectal cancer; PFS, progression-free survival; OS, overall survival; DFS, disease-free survival; RFS, relapse-free survival.

## Conclusions and prospects

At present, malignant tumors with high morbidity and mortality imposed a heavy burden on patients worldwide. Accumulative studies have been trying to reveal the etiology of tumor initiation and explore significant therapies. However, the mechanisms of tumorigenesis, recurrence, metastasis, and drug resistance remained unclear. Researchers reported that the expression of miR-1258 was considerably downregulated in tumor tissues and cell lines. To date, miR-1258 has been shown to act as a tumor suppressor in the development and progression of tumors, suppressing cell cycle transition, metastasis, stemness, migration, invasion, EMT, and glycolysis while boosting cell apoptosis and chemosensitivity. Furthermore, studies revealed that miR-1258 can be employed as a biomarker for early diagnosis and prognosis prediction in tumor patients.

In summary, with the deepening related research of miR-1258, the mechanism of miR-1258 in tumorigenesis and progression would be gradually disclosed. miR-1258 can be exploited as an indicator for early tumor diagnosis and prognosis as well as a potential target for tumor treatment, providing novel perspectives and orientations for precision therapy.

## Author contributions

MQ and YC generated this topic. MQ and YX wrote the manuscript. MQ and GZ searched and collected all relevant literature. MQ and HY constructed the tables and figures. YC supervised and modified the manuscript. MQ and YX contributed equally to this study. All authors contributed to the article and approved the submitted version.
