# Corrigendum: Research progress on microRNA-1258 in the development of human cancer

**DOI:** 10.3389/fonc.2022.1117462

**Published:** 2023-02-02

**Authors:** Mengjia Qian, Yuke Xia, Gong Zhang, Han Yu, Yiyao Cui

**Affiliations:** Department of Thyroid and Breast Surgery, The Affiliated JiangNing Hospital of Nanjing Medical University, Nanjing, China

**Keywords:** miR-1258, cancer, tumor suppressor, biological function, clinical application

**Incorrect Reference**

In the published article, the reference for (1-73) was incorrectly written as [1. Sadreddini S, Baradaran B, Aghebati-Maleki A, Sadreddini S, Shanehbandi D, Fotouhi A, et al. Immune checkpoint blockade opens a new way to cancer immunotherapy. *J Cell Physiol* (2019) 234(6):8541–9. doi: 10.1002/jcp.27816

2. Yang K, Li J, Sun Z, Zhao L, Bai C. Retreatment with immune checkpoint inhibitors in solid tumors: A systematic review. *Ther Adv Med Oncol* (2020) 12:1758835920975353. doi: 10.1177/1758835920975353

3. Inthagard J, Edwards J, Roseweir AK. Immunotherapy: Enhancing the efficacy of this promising therapeutic in multiple cancers. *Clin Sci (Lond)* (2019) 133(2):181–93. doi: 10.1042/CS20181003

4. Suresh K, Naidoo J, Lin CT, Danoff S. Immune checkpoint immunotherapy for non-small cell lung cancer: Benefits and pulmonary toxicities. *Chest* (2018) 154 (6):1416–23. doi: 10.1016/j.chest.2018.08.1048

5. Fan Y, Xie W, Huang H, Wang Y, Li G, Geng Y, et al. Association of immune related adverse events with efficacy of immune checkpoint inhibitors and overall survival in cancers: A systemic review and meta-analysis. *Front Oncol* (2021) 11:633032. doi: 10.3389/fonc.2021.633032

6. Shekarian T, Valsesia-Wittmann S, Caux C, Marabelle A. Paradigm shift in oncology: Targeting the immune system rather than cancer cells. *Mutagenesis* (2015) 30(2):205–11. doi: 10.1093/mutage/geu073

7. Gan J, Huang Y, Fang W, Zhang L. Research progress in immune checkpoint inhibitors for lung cancer in China. *Ther Adv Med Oncol* (2021) 13:17588359211029826. doi: 10.1177/17588359211029826

8. Platten M, von Knebel Doeberitz N, Oezen I, Wick W, Ochs K. Cancer immunotherapy by targeting Ido1/Tdo and their downstream effectors. *Front Immunol* (2014) 5:673. doi: 10.3389/fimmu.2014.00673

9. Hoffmann D, Pilotte L, Stroobant V, Van den Eynde BJ. Induction of tryptophan 2,3-dioxygenase expression in human monocytic Leukemia/Lymphoma cell lines thp-1 and U937. *Int J Tryptophan Res* (2019) 12:1178646919891736. doi: 10.1177/1178646919891736

10. Hanahan D, Weinberg RA. Hallmarks of cancer: The next generation. *Cell* (2011) 144(5):646–74. doi: 10.1016/j.cell.2011.02.013

11. Pilotte L, Larrieu P, Stroobant V, Colau D, Dolusic E, Frederick R, et al. Reversal of tumoral immune resistance by inhibition of tryptophan 2,3-dioxygenase. *Proc Natl Acad Sci U.S.A.* (2012) 109(7):2497–502. doi: 10.1073/pnas.1113873109

12. Hoffmann D, Dvorakova T, Stroobant V, Bouzin C, Daumerie A, Solvay M, et al. Tryptophan 2,3-dioxygenase expression identified in human hepatocellular carcinoma cells and in intratumoral pericytes of most cancers. *Cancer Immunol Res* (2020) 8(1):19–31. doi: 10.1158/2326-6066.CIR-19-0040

13. Dolsak A, Gobec S, Sova M. Indoleamine and tryptophan 2,3-dioxygenases as important future therapeutic targets. *Pharmacol Ther* (2021) 221:107746.doi: 10.1016/j.pharmthera.2020.107746

14. Tina E, Prosen S, Lennholm S, Gasparyan G, Lindberg M, Gothlin Eremo A. Expression profile of the amino acid transporters Slc7a5, Slc7a7, Slc7a8 and the enzyme Tdo2 in basal cell carcinoma. *Br J Dermatol* (2019) 180(1):130–40. doi: 10.1111/bjd.16905

15. Greene LI, Bruno TC, Christenson JL, D’Alessandro A, Culp-Hill R, Torkko K, et al. A role for tryptophan-2,3-Dioxygenase in Cd8 T-cell suppression and evidence of tryptophan catabolism in breast cancer patient plasma. *Mol Cancer Res* (2019) 17(1):131–9. doi: 10.1158/1541-7786.MCR-18-0362

16. Smith LP, Bitler BG, Richer JK, Christenson JL. Tryptophan catabolism in epithelial ovarian carcinoma. *Trends Cancer Res* (2019) 14:1–9.

17. Liu Q, Zhai J, Kong X, Wang X, Wang Z, Fang Y, et al. Comprehensive analysis of the expression and prognosis for Tdo2 in breast cancer. *Mol Ther Oncolytics* (2020) 17:153–68. doi: 10.1016/j.omto.2020.03.013

18. Terai M, Londin E, Rochani A, Link E, Lam B, Kaushal G, et al. Expression of tryptophan 2,3-dioxygenase in metastatic uveal melanoma. *Cancers (Basel)* (2020) 12(2):405. doi: 10.3390/cancers12020405

19. Wang CY, Chiao CC, Phan NN, Li CY, Sun ZD, Jiang JZ, et al. Gene signatures and potential therapeutic targets of amino acid metabolism in estrogen receptor-positive breast cancer. *Am J Cancer Res* (2020) 10(1):95–113.

20. Iwasaki T, Kohashi K, Toda Y, Ishihara S, Yamada Y, Oda Y. Association of pd-L1 and Ido1 expression with jak-stat pathway activation in soft-tissue leiomyosarcoma. *J Cancer Res Clin Oncol* (2021) 147(5):1451–63. doi: 10.1007/s00432-020-03390-9

21. Liberati A, Altman DG, Tetzlaff J, Mulrow C, Gotzsche PC, Ioannidis JP, et al. The prisma statement for reporting systematic reviews and meta-analyses of studies that evaluate healthcare interventions: Explanation and elaboration. *BMJ* (2009) 339:b2700. doi: 10.1136/bmj.b2700

22. Tierney JF, Stewart LA, Ghersi D, Burdett S, Sydes MR. Practical methods for incorporating summary time-to-Event data into meta-analysis. *Trials* (2007) 8:16. doi: 10.1186/1745-6215-8-16

23. Stang A. Critical evaluation of the Newcastle-Ottawa scale for the assessment of the quality of nonrandomized studies in meta-analyses. *Eur J Epidemiol* (2010) 25(9):603–5. doi: 10.1007/s10654-010-9491-z

24. Yusuf S, Peto R, Lewis J, Collins R, Sleight P. Beta blockade during and after myocardial infarction: An overview of the randomized trials. *Prog Cardiovasc Dis* (1985) 27(5):335–71. doi: 10.1016/s0033-0620(85)80003-7

25. Zintzaras E, Ioannidis JP. Hegesma: Genome search meta-analysis and heterogeneity testing. *Bioinformatics* (2005) 21(18):3672–3. doi: 10.1093/bioinformatics/bti536

26. Sterne JA, Egger M. Funnel plots for detecting bias in meta-analysis: Guidelines on choice of axis. *J Clin Epidemiol* (2001) 54(10):1046–55. doi: 10.1016/s0895-4356(01)00377-8

27. Egger M, Davey Smith G, Schneider M, Minder C. Bias in meta-analysis detected by a simple, graphical test. *BMJ* (1997) 315(7109):629–34. doi: 10.1136/bmj.315.7109.629

28. Tang Z, Kang B, Li C, Chen T, Zhang Z. Gepia2: An enhanced web server for Large-scale expression profiling and interactive analysis. *Nucleic Acids Res* (2019) 47(W1):W556–W60. doi: 10.1093/nar/gkz430

29. Liao Y, Wang J, Jaehnig EJ, Shi Z, Zhang B. Webgestalt 2019: Gene set analysis toolkit with revamped uis and apis. *Nucleic Acids Res* (2019) 47(W1): W199–205. doi: 10.1093/nar/gkz401

30. Sumitomo M, Takahara K, Zennami K, Nagakawa T, Maeda Y, Shiogama K, et al. Tryptophan 2,3-dioxygenase in tumor cells is associated with resistance to immunotherapy in renal cell carcinoma. *Cancer Sci* (2021) 112(3):1038–47. doi: 10.1111/cas.14797

31. Du L, Xing Z, Tao B, Li T, Yang D, Li W, et al. Both Ido1 and tdo contribute to the malignancy of gliomas *Via* the kyn-Ahr-Aqp4 signaling pathway. *Signal Transduct Target Ther* (2020) 5(1):10. doi: 10.1038/s41392-019-0103-4

32. de Hosson LD, Takkenkamp TJ, Kats-Ugurlu G, Bouma G, Bulthuis M, de Vries EGE, et al. Neuroendocrine tumours and their microenvironment. *Cancer Immunol Immunother* (2020) 69(8):1449–59. doi: 10.1007/s00262-020-02556-1

33. Chen X, Zang Y, Li D, Guo J, Wang Y, Lin Y, et al. Ido, tdo, and ahr overexpression is associated with poor outcome in diffuse Large b-cell lymphoma patients in the rituximab era. *Med (Baltimore)* (2020) 99(21):e19883. doi: 10.1097/MD.0000000000019883

34. Pham QT, Oue N, Sekino Y, Yamamoto Y, Shigematsu Y, Sakamoto N, et al. Tdo2 overexpression is associated with cancer stem cells and poor prognosis in esophageal squamous cell carcinoma. *Oncology* (2018) 95(5):297–308. doi: 10.1159/000490725

35. Chen IC, Lee KH, Hsu YH, Wang WR, Chen CM, Cheng YW. Expression pattern and clinicopathological relevance of the indoleamine 2,3-dioxygenase 1/Tryptophan 2,3-dioxygenase protein in colorectal cancer. *Dis Markers* (2016) 2016:8169724. doi: 10.1155/2016/8169724

36. Li S, Li L, Wu J, Song F, Qin Z, Hou L, et al. Tdo promotes hepatocellular carcinoma progression. *Onco Targets Ther* (2020) 13:5845–55. doi: 10.2147/OTT.S252929

37. Wardhani LO, Matsushita M, Iwasaki T, Kuwamoto S, Nonaka D, Nagata K, et al. Expression of the Ido1/Tdo2-ahr pathway in tumor cells or the tumor microenvironment is associated with merkel cell polyomavirus status and prognosis in merkel cell carcinoma. *Hum Pathol* (2019) 84:52–61. doi: 10.1016/j.humpath.2018.09.003

38. Riess C, Schneider B, Kehnscherper H, Gesche J, Irmscher N, Shokraie F, et al. Activation of the kynurenine pathway in human malignancies can be suppressed by the cyclin-dependent kinase inhibitor dinaciclib. *Front Immunol* (2020) 11:55. doi: 10.3389/fimmu.2020.00055

39. Theate I, van Baren N, Pilotte L, Moulin P, Larrieu P, Renauld JC, et al. Extensive profiling of the expression of the indoleamine 2,3-dioxygenase 1 protein in normal and tumoral human tissues. *Cancer Immunol Res* (2015) 3(2):161–72. doi: 10.1158/2326-6066.CIR-14-0137

40. Marszalek-Grabska M, Walczak K, Gawel K, Wicha-Komsta K, Wnorowska S, Wnorowski A, et al. Kynurenine emerges from the shadows - current knowledge on its fate and function. *Pharmacol Ther* (2021) 225:107845. doi: 10.1016/j.pharmthera.2021.107845

41. Kim M, Tomek P. Tryptophan: A rheostat of cancer immune escape mediated by immunosuppressive enzymes Ido1 and tdo. *Front Immunol* (2021) 12:636081. doi: 10.3389/fimmu.2021.636081

42. D’Amato NC, Rogers TJ, Gordon MA, Greene LI, Cochrane DR, Spoelstra NS, et al. A Tdo2-ahr signaling axis facilitates anoikis resistance and metastasis in triple-negative breast cancer. *Cancer Res* (2015) 75(21):4651–64. doi: 10.1158/0008-5472.CAN-15-2011

43. Chen LB, Zhu SP, Liu TP, Zhao H, Chen PF, Duan YJ, et al. Cancer associated fibroblasts promote renal cancer progression through a Tdo/Kyn/Ahr dependent signaling pathway. *Front Oncol* (2021) 11:628821. doi: 10.3389/fonc.2021.628821

44. Paccosi S, Cecchi M, Silvano A, Fabbri S, Parenti A. Different effects of tryptophan 2,3-dioxygenase inhibition on sk-Mel-28 and hct-8 cancer cell lines. *J Cancer Res Clin Oncol* (2020) 146(12):3155–63. doi: 10.1007/s00432-020-03351-2

45. Li L, Wang T, Li S, Chen Z, Wu J, Cao W, et al. Tdo2 promotes the emt of hepatocellular carcinoma through kyn-ahr pathway. *Front Oncol* (2020) 10:562823. doi: 10.3389/fonc.2020.562823

46. Opitz CA, Litzenburger UM, Sahm F, Ott M, Tritschler I, Trump S, et al. An endogenous tumour-promoting ligand of the human aryl hydrocarbon receptor. *Nature* (2011) 478(7368):197–203. doi: 10.1038/nature10491

47. Reed MR, Maddukuri L, Ketkar A, Byrum SD, Zafar MK, Bostian ACL, et al. Inhibition of tryptophan 2,3-dioxygenase impairs DNA damage tolerance and repair in glioma cells. *NAR Cancer* (2021) 3(2):zcab014. doi: 10.1093/narcan/zcab014

48. Boros FA, Vecsei L. Immunomodulatory effects of genetic alterations affecting the kynurenine pathway. *Front Immunol* (2019) 10:2570. doi: 10.3389/fimmu.2019.02570

49. Campesato LF, Budhu S, Tchaicha J, Weng CH, Gigoux M, Cohen IJ, et al. Blockade of the ahr restricts a treg-macrophage suppressive axis induced by lkynurenine. *Nat Commun* (2020) 11(1):4011. doi: 10.1038/s41467-020-17750-z

50. Holmgaard RB, Zamarin D, Li Y, Gasmi B, Munn DH, Allison JP, et al. Tumor-expressed ido recruits and activates mdscs in a treg-dependent manner. *Cell Rep* (2015) 13(2):412–24. doi: 10.1016/j.celrep.2015.08.077

51. Gurczynski SJ, Pereira NL, Hrycaj SM, Wilke C, Zemans RL, Moore BB. Stem cell transplantation uncovers tdo-ahr regulation of lung dendritic cells in herpesvirus-induced pathology. *JCI Insight* (2021) 6(2):e139965. doi: 10.1172/jci.insight.139965

52. Qin Y, Wang N, Zhang X, Han X, Zhai X, Lu Y. Ido and tdo as a potential therapeutic target in different types of depression. *Metab Brain Dis* (2018) 33(6):1787–800. doi: 10.1007/s11011-018-0290-7

53. Perez-Castro L, Garcia R, Venkateswaran N, Barnes S, Conacci-Sorrell M. Tryptophan and its metabolites in normal physiology and cancer etiology. *FEBS J* (2021). doi: 10.1111/febs.16245

54. Jia YQ, Yang B, Wen LL, Mu WX, Wang Z, Cheng B. Prognostic value of immune checkpoint molecules in head and neck cancer: A meta-analysis. *Aging (Albany NY)* (2019) 11(2):501–22. doi: 10.18632/aging.101756

55. Yu CP, Fu SF, Chen X, Ye J, Ye Y, Kong LD, et al. The clinicopathological and prognostic significance of Ido1 expression in human solid tumors: Evidence from a systematic review and meta-analysis. *Cell Physiol Biochem* (2018) 49(1):134–43. doi: 10.1159/000492849

56. Wang S, Wu J, Shen H, Wang J. The prognostic value of ido expression in solid tumors: A systematic review and meta-analysis. *BMC Cancer* (2020) 20(1):471. doi: 10.1186/s12885-020-06956-5

57. Chen S, Tan J, Zhang A. The ups, downs and new trends of Ido1 inhibitors. *Bioorg Chem* (2021) 110:104815. doi: 10.1016/j.bioorg.2021.104815

58. Liu M, Wang X, Wang L, Ma X, Gong Z, Zhang S, et al. Targeting the Ido1 pathway in cancer: From bench to bedside. *J Hematol Oncol* (2018) 11(1):100. doi: 10.1186/s13045-018-0644-y

59. Fiore A, Murray PJ. Tryptophan and indole metabolism in immune regulation. *Curr Opin Immunol* (2021) 70:7–14. doi: 10.1016/j.coi.2020.12.001

60. Muller AJ, Manfredi MG, Zakharia Y, Prendergast GC. Inhibiting ido pathways to treat cancer: Lessons from the echo-301 trial and beyond. *Semin Immunopathol* (2019) 41(1):41–8. doi: 10.1007/s00281-018-0702-0

61. Cui G, Lai F, Wang X, Chen X, Xu B. Design, synthesis and biological evaluation of indole-2-Carboxylic acid derivatives as Ido1/Tdo dual inhibitors. *Eur J Med Chem* (2020) 188:111985. doi: 10.1016/j.ejmech.2019.111985

62. Feng X, Shen P, Wang Y, Li Z, Bian J. Synthesis and *in vivo* antitumor evaluation of an orally active potent phosphonamidate derivative targeting Ido1/Ido2/Tdo. *Biochem Pharmacol* (2019) 168:214–23. doi: 10.1016/j.bcp.2019.07.011

63. Pei Z, Mendonca R, Gazzard L, Pastor R, Goon L, Gustafson A, et al. Aminoisoxazoles as potent inhibitors of tryptophan 2,3-dioxygenase 2 (Tdo2). *ACS Med Chem Lett* (2018) 9(5):417–21. doi: 10.1021/acsmedchemlett.7b00427

64. Menke A. Is the hpa axis as target for depression outdated, or is there a new hope? *Front Psychiatry* (2019) 10:101. doi: 10.3389/fpsyt.2019.00101

65. Hua S, Wang X, Chen F, Gou S. Novel conjugates with dual suppression of glutathione s-transferases and tryptophan-2,3-Dioxygenase activities for improving hepatocellular carcinoma therapy. *Bioorg Chem* (2019) 92:103191. doi: 10.1016/j.bioorg.2019.103191

66. Andersen MH. The targeting of tumor-associated macrophages by vaccination. *Cell Stress* (2019) 3(5):139–40. doi: 10.15698/cst2019.05.185

67. Platten M, Nollen EAA, Rohrig UF, Fallarino F, Opitz CA. Tryptophan metabolism as a common therapeutic target in cancer, neurodegeneration and beyond. *Nat Rev Drug Discov* (2019) 18(5):379–401. doi: 10.1038/s41573-019-0016-5

68. Hua S, Chen F, Wang X, Wang Y, Gou S. Pt(Iv) hybrids containing a tdo inhibitor serve as potential anticancer immunomodulators. *J Inorg Biochem* (2019) 195:130–40. doi: 10.1016/j.jinorgbio.2019.02.004

69. Zhou Q, Shi Y, Chen C, Wu F, Chen Z. A narrative review of the roles of indoleamine 2,3-dioxygenase and tryptophan-2,3-Dioxygenase in liver diseases. *Ann Transl Med* (2021) 9(2):174. doi: 10.21037/atm-20-3594

70. Ye Z, Yue L, Shi J, Shao M, Wu T. Role of ido and tdo in cancers and related diseases and the therapeutic implications. *J Cancer* (2019) 10(12):2771–82. doi: 10.7150/jca.31727

71. Li Y, Zhang S, Wang R, Cui M, Liu W, Yang Q, et al. Synthesis of novel tryptanthrin derivatives as dual inhibitors of indoleamine 2,3-dioxygenase 1 and tryptophan 2,3-dioxygenase. *Bioorg Med Chem Lett* (2020) 30(11):127159. doi: 10.1016/j.bmcl.2020.127159

72. Lovelace MD, Varney B, Sundaram G, Lennon MJ, Lim CK, Jacobs K, et al. Recent evidence for an expanded role of the kynurenine pathway of tryptophan metabolism in neurological diseases. *Neuropharmacology* (2017) 112(Pt B):373–88. doi: 10.1016/j.neuropharm.2016.03.024

73. Badawy AA. Tryptophan: The key to boosting brain serotonin synthesis in depressive illness. J Psychopharmacol (2013) 27(10):878–93. doi: 10.1177/0269881113499209].

It should be [1. Ambros V. microRNAs: tiny regulators with great potential. *Cell* (2001) 107(7):823-826. doi: 10.1016/s0092-8674(01)00616-x

2. Gregory RI, Chendrimada TP, Cooch N, Shiekhattar R. Human RISC couples microRNA biogenesis and posttranscriptional gene silencing. Cell (2005) 123(4):631-640. doi: 10.1016/j.cell.2005.10.022

3. Lee, Y, Ahn, C, Han, J, Choi, H, Kim, J, Yim, J, et al. The nuclear RNase III Drosha initiates microRNA processing. *Nature* (2003) 425(6956):415-419. doi: 10.1038/nature01957

4. Ke XS, Liu CM, Liu DP, Liang CC. MicroRNAs: key participants in gene regulatory networks. *Current Opinion in Chemical Biology* (2003) 7(4):516-523. doi: 10.1016/s1367-5931(03)00075-9

5. Tsuchiya S, Okuno Y, Tsujimoto G. MicroRNA: Biogenetic and functional mechanisms and involvements in cell differentiation and cancer. *J Pharmacological Sciences* (2006) 101(4):267-270. doi: 10.1254/jphs.cpj06013x

6. Liu J. Control of protein synthesis and mRNA degradation by microRNAs. *Current Opinion Cell Biology* (2008) 20(2):214-221. doi: 10.1016/j.ceb.2008.01.006

7. Hu T, Shen H, Li J, Yang P, Gu Q, Fu Z. RFC2, a direct target of miR-744, modulates the cell cycle and promotes the proliferation of CRC cells. *J Cellular Physiology* (2020) 235(11):8319-8333. doi: 10.1002/jcp.29676

8. Qian, W, Feng, Y, Li, J, Peng, W, Gu, Q, Zhang, Z, et al. Construction of ceRNA networks reveals differences between distal and proximal colon cancers. *Oncol Reports* (2019) 41(5):3027-3040. doi: 10.3892/or.2019.7083

9. Chen, F, Chu, L, Li, J, Shi, Y, Xu, B, Gu, J, et al. Hypoxia induced changes in miRNAs and their target mRNAs in extracellular vesicles of esophageal squamous cancer cells. *Thoracic Cancer* (2020) 11(3):570-580. doi: 10.1111/1759-7714.13295

10. Ma, M, Li, J, Zhang, Z, Sun, J, Liu, Z, Zeng, Z, et al. The Role and Mechanism of microRNA-1224 in Human Cancer. *Front Oncol* (2022) 12:858892. doi: 10.3389/fonc.2022.858892

11. Li, J, Sun, J, Liu, Z, Zeng, Z, Ouyang, S, Zhang, Z, et al. The Roles of Non-Coding RNAs in Radiotherapy of Gastrointestinal Carcinoma. *Front Cell and Developmental Biology* (2022) 10:862563. doi: 10.3389/fcell.2022.862563

12. Li, J, Feng, Y, Heng, D, Chen, R, Wang, Y, Xu, Z, et al. Circulating non-coding RNA cluster predicted the tumorigenesis and development of colorectal carcinoma. *Aging* (2020) 12(22):23047-23066. doi: 10.18632/aging.104055

13. Chen, F, Xu, B, Li, J, Yang, X, Gu, J, Yao, X, et al. Hypoxic tumour cell-derived exosomal miR-340-5p promotes radioresistance of oesophageal squamous cell carcinoma *via* KLF10. *J Experimental & Clin Cancer Res: CR* (2021) 40(1):38. doi: 10.1186/s13046-021-01834-9

14. Zhang, Y, Peng, C, Li, J, Zhang, D, Zhang, C, Jin, K, et al. Long non-coding RNA CCDC144NL-AS1 promotes cell proliferation by regulating the miR-363-3p/GALNT7 axis in colorectal cancer. *J Cancer* (2022) 13(3):752-763. doi: 10.7150/jca.65885

15. Li, J, Han, X, Gu, Y, Wu, J, Song, J, Shi, Z, et al.. LncRNA MTX2-6 Suppresses Cell Proliferation by Acting as ceRNA of miR-574-5p to Accumulate SMAD4 in Esophageal Squamous Cell Carcinoma. *Front Cell Developmental B* (2021) 9:654746. doi: 10.3389/fcell.2021.654746

16. Zhang, Z, Wang, S, Ji, D, Qian, W, Wang, Q, Li, J, et al. Construction of a ceRNA network reveals potential lncRNA biomarkers in rectal adenocarcinoma. *Oncol Reports* (2018) 39(5):2101-2113. doi: 10.3892/or.2018.6296

17. Zhang Y, Kong X, Zhang J, Wang X. Functional Analysis of Bronchopulmonary Dysplasia-Related Neuropeptides in Preterm Infants and miRNA-Based Diagnostic Model Construction. *Computational Mathematical Methods Med* (2022) 2022:5682599. doi: 10.1155/2022/5682599

18. Yan, Q, Ma, X, Shen, C, Cao, X, Feng, N, Qin, D, et al. Inhibition of Kaposi’s sarcoma-associated herpesvirus lytic replication by HIV-1 Nef and cellular microRNA hsa-miR-1258. *J Virol* (2014) 88(9):4987-5000. doi: 10.1128/jvi.00025-14

19. Yang X, Gao Y, Huang S, Su C, Wang J, Zheng N. Whole transcriptome-based ceRNA network analysis revealed ochratoxin A-induced compromised intestinal tight junction proteins through WNT/Ca(2+) signaling pathway. *Ecotoxicology Environmental Safety* (2021) 224:112637. doi: 10.1016/j.ecoenv.2021.112637

20. Cao, Y, Deng, B, Zhang, S, Gao, H, Song, P, Zhang, J, et al.. Astragalus polysaccharide regulates brown adipogenic differentiation through miR-1258-5p-modulated cut-like homeobox 1 expression. *Acta Biochimica et Biophysica Sinica* (2021) 53(12):1713-1722. doi: 10.1093/abbs/gmab151

21. Fang Q, Liu H, Zhou A, Zhou H, Zhang Z. Circ_0046599 Promotes the Development of Hepatocellular Carcinoma by Regulating the miR-1258/RPN2 Network. *Cancer Management Res* (2020) 12:6849-6860. doi: 10.2147/cmar.S253510

22. Zhang D, Zhang Y, Zhang X, Zhai H, Sun X, Li Y. Circ_0046600 promotes hepatocellular carcinoma progression *via* up-regulating SERBP1 through sequestering miR-1258. *Pathol Res P* (2021) 228:153681. doi: 10.1016/j.prp.2021.153681

23. Lin, W, Lin, J, Li, J, Lin, Y, Chen, S, Wu, Y, et al. Kindlin-2-miR-1258-TCF4 feedback loop promotes hepatocellular carcinoma invasion and metastasis. *J Gastroenterol* (2022) 57(5):372-386. doi: 10.1007/s00535-022-01866-8

24. Hu, M, Wang, M, Lu, H, Wang, X, Fang, X, Wang, J, et al. Loss of miR-1258 contributes to carcinogenesis and progression of liver cancer through targeting CDC28 protein kinase regulatory subunit 1B. *Oncotarget* (2016) 7(28):43419-43431. doi: 10.18632/oncotarget.9728

25. Huang, WJ, Tian, XP, Bi, SX, Zhang, SR, He, TS, Song, LY, et al. The β-catenin/TCF-4-LINC01278-miR-1258-Smad2/3 axis promotes hepatocellular carcinoma metastasis. *Oncogene* (2020) 39(23):4538-4550. doi: 10.1038/s41388-020-1307-3

26. Shi, J, Chen, P, Sun, J, Song, Y, Ma, B, Gao, P, et al. MicroRNA-1258: An invasion and metastasis regulator that targets heparanase in gastric cancer. *Oncol Letters* (2017) 13(5):3739-3745. doi: 10.3892/ol.2017.5886

27. Zhang W, Wu G, Sun P, Zhu Y, Zhang H. circ_SMAD2 regulate colorectal cancer cells proliferation through targeting miR-1258/RPN2 signaling pathway. *J Cancer* (2021) 12(6):1678-1686. doi: 10.7150/jca.50888

28. Zhang, Z, Li, J, Huang, Y, Peng, W, Qian, W, Gu, J, et al. Upregulated miR-1258 regulates cell cycle and inhibits cell proliferation by directly targeting E2F8 in CRC. *Cell Proliferation* (2018) 51(6):e12505. doi: 10.1111/cpr.12505

29. Hwang, JS, Jeong, EJ, Choi, J, Lee, YJ, Jung, E, Kim, SK, et al. MicroRNA-1258 Inhibits the Proliferation and Migration of Human Colorectal Cancer Cells through Suppressing CKS1B Expression. *Genes* (2019) 10(11). doi: 10.3390/genes10110912

30. Zhang H, Jiang S, Guo L, Li X. MicroRNA-1258, regulated by c-Myb, inhibits growth and epithelial-to-mesenchymal transition phenotype *via* targeting SP1 in oral squamous cell carcinoma. *J Cellular Molecular Medicine* (2019) 23(4):2813-2821. doi: 10.1111/jcmm.14189

31. Jia Z, Wang PS, Yang Y, Zhu DY, Wang ZH, Wang W. [LncRNA ASB16-AS1 regulates the proliferation, migration and invasion of esophageal cancer cells by targeting miR-1258]. *Zhonghua zhong liu za zhi [Chinese journal of oncology]* (2021) 43(7):762-768. doi: 10.3760/cma.j.cn112152-20200509-00430

32. Jiang, W, Wei, K, Pan, C, Li, H, Cao, J, Han, X, et al. MicroRNA-1258 suppresses tumour progression *via* GRB2/Ras/Erk pathway in non-small-cell lung cancer. *Cell proliferation* (2018) 51(6):e12502. doi: 10.1111/cpr.12502

33. Liu H, Chen X, Gao W, Jiang G. The expression of heparanase and microRNA-1258 in human non-small cell lung cancer. *Tumour biology: the journal of the International Society for Oncodevelopmental Biology and Medicine* (2012) 33(5):1327-1334. doi: 10.1007/s13277-012-0380-9

34. Wang R, Liu H, Dong M, Huang D, Yi J. Exosomal hsa_circ_0000519 modulates the NSCLC cell growth and metastasis *via* miR-1258/RHOV axis. *Open medicine (Warsaw, Poland)* (2022) 17(1):826-840. doi: 10.1515/med-2022-0428

35. Li W, Yang X, Shi C, Zhou Z. Hsa_circ_002178 Promotes the Growth and Migration of Breast Cancer Cells and Maintains Cancer Stem-like Cell Properties Through Regulating miR-1258/KDM7A Axis. *Cell transplantation* (2020) 29:963689720960174. doi: 10.1177/0963689720960174

36. Sang, M, Li, A, Wang, X, Chen, C, Liu, K, Bai, L, et al. Identification of three miRNAs signature as a prognostic biomarker in breast cancer using bioinformatics analysis. *Translational Cancer Research* (2020) 9(3):1884-1893. doi: 10.21037/tcr.2020.02.21

37. Zhao X. miR-1258 Regulates Cell Proliferation and Cell Cycle to Inhibit the Progression of Breast Cancer by Targeting E2F1. *BioMed Research International* (2020) 2020:1480819. doi: 10.1155/2020/1480819

38. Loginov VI, Burdennyy AM, Pronina IV, et al. [Novel miRNA genes hypermethylated in breast cancer]. *Molekuliarnaia biologiia* (2016) 50(5):797-802. doi: 10.7868/s0026898416050104

39. Tang, D, Zhang, Q, Zhao, S, Wang, J, Lu, K, Song, Y, et al. The expression and clinical significance of microRNA-1258 and heparanase in human breast cancer. *Clinical Biochemistr*y (2013) 46(10-11):926-932. doi: 10.1016/j.clinbiochem.2013.01.027

40. Zhang L, Sullivan PS, Goodman JC, Gunaratne PH, Marchetti D. MicroRNA-1258 suppresses breast cancer brain metastasis by targeting heparanase. *Cancer Research* (2011) 71(3):645-654. doi: 10.1158/0008-5472.Can-10-1910

41. Peng X, Zhang Y, Gao J, Cai C. MiR-1258 promotes the apoptosis of cervical cancer cells by regulating the E2F1/P53 signaling pathway. *Experimental and Molecular Pathology* (2020) 114:104368. doi: 10.1016/j.yexmp.2020.104368

42. Wang LQ, Kumar S, Calin GA, Li Z, Chim CS. Frequent methylation of the tumour suppressor miR-1258 targeting PDL1: implication in multiple myeloma-specific cytotoxicity and prognostification. *British Journal of Haematology* (2020) 190(2):249-261. doi: 10.1111/bjh.16517

43. Wang LJ, Cai HQ. miR-1258: a novel microRNA that controls TMPRSS4 expression is associated with malignant progression of papillary thyroid carcinoma. *Endokrynologia Polska* (2020) 71(2):146-152. doi: 10.5603/EP.a2020.0009

44. Qin, H, Gui, Y, Ma, R, Zhang, H, Guo, Y, Ye, Y, et al. miR-1258 Attenuates Tumorigenesis Through Targeting E2F1 to Inhibit PCNA and MMP2 Transcription in Glioblastoma. *Frontiers in Oncology* (2021) 11:671144. doi: 10.3389/fonc.2021.671144

45. Liu, W, Zhou, Z, Zhang, Q, Rong, Y, Li, L, Luo, Y, et al. Overexpression of miR-1258 inhibits cell proliferation by targeting AKT3 in osteosarcoma. *Biochemical and Biophysical Research Communications* (2019) 510(3):479-486. doi: 10.1016/j.bbrc.2019.01.139

46. Braga, EA, Loginov, VI, Burdennyi, AM, Filippova, EA, Pronina, IV, Kurevlev, SV, et al. Five Hypermethylated MicroRNA Genes as Potential Markers of Ovarian Cancer. *Bulletin of experimental biology and medicine* (2018) 164(3):351-355. doi: 10.1007/s10517-018-3988-y

47. Filippova, EA, Loginov, VI, Burdennyi, AM, Braga, EA, Pronina, IV, Kazubskaya, TP, et al. Hypermethylated Genes of MicroRNA in Ovarian Carcinoma: Metastasis Prediction Marker Systems. *Bulletin of Experimental Biology and Medicine* (2019) 167(1):79-83. doi: 10.1007/s10517-019-04465-5

48. Loginov, VI, Burdennyy, AM, Filippova, EA, Pronina, IV, Kazubskaya, TP, Kushlinsky, DN, et al. [Hypermethylation of miR-107, miR-130b, miR-203a, miR-1258 Genes Associated with Ovarian Cancer Development and Metastasis]. *Molekuliarnaia Biologiia* (2018) 52(5):801-809. doi: 10.1134/s0026898418050105

49. Torres-Ferreira, J, Ramalho-Carvalho, J, Gomez, A, Menezes, FD, Freitas, R, Oliveira, J, et al. MiR-193b promoter methylation accurately detects prostate cancer in urine sediments and miR-34b/c or miR-129-2 promoter methylation define subsets of clinically aggressive tumors. *Molecular Cancer* (2017) 16(1):26. doi: 10.1186/s12943-017-0604-0

50. Zhou, R, Jia, W, Gao, X, Deng, F, Fu, K, Zhao, T, et al. CircCDYL Acts as a Tumor Suppressor in Wilms’ Tumor by Targeting miR-145-5p. *Frontiers in Cell and Developmental Biology* (2021) 9:668947. doi: 10.3389/fcell.2021.668947

51. Qi X, Chen X, Zhao Y, Chen J, Niu B, Shen B. Prognostic Roles of ceRNA Network-Based Signatures in Gastrointestinal Cancers. *Frontiers in Oncology* (2022) 12:921194. doi: 10.3389/fonc.2022.921194

52. Guo, L, Jia, L, Luo, L, Xu, X, Xiang, Y, Ren, Y, et al. Critical Roles of Circular RNA in Tumor Metastasis *via* Acting as a Sponge of miRNA/isomiR. *International Journal of Molecular Sciences* (2022) 23(13). doi: 10.3390/ijms23137024

53. Boman BM, Wicha MS. Cancer stem cells: a step toward the cure. *Journal of Clinical Oncology: Official Journal of the American Society of Clinical Oncology* (2008) 26(17):2795-2799. doi: 10.1200/jco.2008.17.7436

54. Ming D, Zhang S, Liu X, Xu C, Zhang X. Nondiploid cancer cells: Stress, tolerance and therapeutic inspirations. *Biochimica et Biophysica Acta Reviews on Cancer* (2022) 2022:188794. doi: 10.1016/j.bbcan.2022.188794

55. Chaturvedi SS, Ramanan R, Waheed SO, Karabencheva-Christova TG, Christov CZ. Structure-function relationships in KDM7 histone demethylases. *Advances in Protein Chemistry and Structural Biology* (2019) 117:113-125. doi: 10.1016/bs.apcsb.2019.08.005

56. Wang J, Li D, Zhao B, Kim J, Sui G, Shi J. Small Molecule Compounds of Natural Origin Target Cellular Receptors to Inhibit Cancer Development and Progression. *International Journal of Molecular Sciences* (2022) 23(5). doi: 10.3390/ijms23052672

57. Yablonski D. Bridging the Gap: Modulatory Roles of the Grb2-Family Adaptor, Gads, in Cellular and Allergic Immune Responses. *Frontiers in Immunology* (2019) 10:1704. doi: 10.3389/fimmu.2019.01704

58. Hu X, Wang J, Chu M, Liu Y, Wang ZW, Zhu X. Emerging Role of Ubiquitination in the Regulation of PD-1/PD-L1 in Cancer Immunotherapy. *Molecular Therapy: The Journal of the American Society of Gene Therapy* (2021) 29(3):908-919. doi: 10.1016/j.ymthe.2020.12.032

59. Zeng Q, Ma X, Song Y, Chen Q, Jiao Q, Zhou L. Targeting regulated cell death in tumor nanomedicines. *Theranostics* (2022) 12(2):817-841. doi: 10.7150/thno.67932

60. Sleeman JP, Thiele W. Tumor metastasis and the lymphatic vasculature. *International Journal of Cancer* (2009) 125(12):2747-2756. doi: 10.1002/ijc.24702

61. Dong, H, Diao, H, Zhao, Y, Xu, H, Pei, S, Gao, J, et al. Overexpression of matrix metalloproteinase-9 in breast cancer cell lines remarkably increases the cell malignancy largely *via* activation of transforming growth factor beta/SMAD signalling. *Cell Proliferation* (2019) 52(5):e12633. doi: 10.1111/cpr.12633

62. Brinckerhoff CE, Matrisian LM. Matrix metalloproteinases: a tail of a frog that became a prince. *Nature Reviews Molecular Cell Biology* (2002) 3(3):207-214. doi: 10.1038/nrm763

63. Diaz-Moralli S, Tarrado-Castellarnau M, Miranda A, Cascante M. Targeting cell cycle regulation in cancer therapy. *Pharmacology & Therapeutics* 2013;138(2):255-271. doi: 10.1016/j.pharmthera.2013.01.011

64. Tsantoulis PK, Gorgoulis VG. Involvement of E2F transcription factor family in cancer. *European Journal of Cancer (Oxford, England: 1990)* (2005) 41(16):2403-2414. doi: 10.1016/j.ejca.2005.08.005

65. Shi W, Huang Q, Xie J, Wang H, Yu X, Zhou Y. CKS1B as Drug Resistance-Inducing Gene-A Potential Target to Improve Cancer Therapy. *Frontiers in Oncology* (2020) 10:582451. doi: 10.3389/fonc.2020.582451

66. Yilmaz M, Christofori G. EMT, the cytoskeleton, and cancer cell invasion. *Cancer Metastasis Reviews* (2009) 28(1-2):15-33. doi: 10.1007/s10555-008-9169-0

67. Lamouille S, Xu J, Derynck R. Molecular mechanisms of epithelial-mesenchymal transition. *Nature Reviews Molecular Cell Biology* (2014) 15(3):178-196. doi: 10.1038/nrm3758

68. Li, J, Peng, W, Yang, P, Chen, R, Gu, Q, Qian, W, et al. MicroRNA-1224-5p Inhibits Metastasis and Epithelial-Mesenchymal Transition in Colorectal Cancer by Targeting SP1-Mediated NF-κB Signaling Pathways. *Frontiers in Oncology* (2020) 10:294. doi: 10.3389/fonc.2020.00294

69. Liu, Y, Song, Y, Cao, M, Fan, W, Cui, Y, Cui, Y, et al. A novel EHD1/CD44/Hippo/SP1 positive feedback loop potentiates stemness and metastasis in lung adenocarcinoma. *Clinical and Translational Medicine* (2022) 12(4):e836. doi: 10.1002/ctm2.836

70. Mayfosh AJ, Nguyen TK, Hulett MD. The Heparanase Regulatory Network in Health and Disease. *International Journal of Molecular Sciences* (2021) 22(20). doi: 10.3390/ijms222011096

71. Kaur R, Deb PK, Diwan V, Saini B. Heparanase Inhibitors in Cancer Progression: Recent Advances. *Current Pharmaceutical Design* (2021) 27(1):43-68. doi: 10.2174/1381612826666201113105250

72. Rupaimoole R, Slack FJ. MicroRNA therapeutics: towards a new era for the management of cancer and other diseases. *Nature reviews Drug Discovery* (2017) 16(3):203-222. doi: 10.1038/nrd.2016.246

73. Sharma GG, Okada Y, Von Hoff D, Goel A. Non-coding RNA biomarkers in pancreatic ductal adenocarcinoma. *Seminars in Cancer Biology* (2020). doi: 10.1016/j.semcancer.2020.10.001

The authors apologize for this error and state that this does not change the scientific conclusions of the article in any way. The original article has been updated.

